# A recombinase system facilitates cloning of expression cassettes in the ciliate *Tetrahymena thermophila*

**DOI:** 10.1186/1471-2180-7-12

**Published:** 2007-03-01

**Authors:** Thomas Weide, Ulrike Bockau, Angelika Rave, Lutz Herrmann, Marcus WW Hartmann

**Affiliations:** 1 Universitaetskliniken Muenster (UKM), Abteilung für Molekulare Nephrologie, Domagkstr. 3a, D-48149 Muenster, Germany; 2 Cilian AG, Johann-Krane-Weg 42, D-48149 Muenster, Germany; 3 Institut für allgemeine Zoologie und Genetik, Universitaet Muenster, Schloßplatz 5, D-48149 Muenster, Germany; 4 Provendis GmbH, Eppinghofer Str. 50, 48468 Muelheim an der Ruhr, Germany

## Abstract

**Background:**

*Tetrahymena thermophila *is one of the best characterized unicellular eukaryotes and its genome is sequenced in its entirety. However, the AT-richness of the genome and an unusual codon usage cause problems in cloning and expression of the ciliate DNA. To overcome these technical hiatuses we developed a *Cre*-dependent recombinase system.

**Results:**

We created novel donor and acceptor vectors that facilitate the transfer of expression cassettes from the donor into novel acceptor plasmid. Expression vectors were used that encode the 19 kDa C-terminus of the MSP1 protein of *Plasmodium falciparum *and a blasticidin S (bsdR) resistance gene, respectively. The functional expression of these genes was demonstrated by western blot analysis with MSP1 specific antibodies and by a blasticidin growing assay.

**Conclusion:**

The *Cre *dependent recombinase system in combination with the modular structure of the donor vectors ease cloning and expression of foreign genes in the ciliate system, providing a powerful tool for protistology research in future.

## Background

The ciliate *Tetrahymena thermophila *has been successfully used as a model system in molecular and cell biology for decades. Fundamental discoveries such as ribozymes, telomeric repeats, telomerases or the function of scan RNAs were first studied in this eukaryotic microorganism [1-9]. In addition, cells grow fast to high cell densities in inexpensive media and simple bioreactor infrastructure and several foreign proteins have been expressed, suggesting that *T. thermophila*has the potential to become an excellent expression host [10-12].

Ciliates characteristically possess two nuclei, a somatic macronucleus (MAC) and a germline micronucleus (MIC)[13]. Recently, the entire genome of the MAC of *T. thermophila *has been characterized[2]. A shotgun sequencing analysis of the MAC revealed that *T. thermophila *is 104 Mb in length and has approximately 225 MAC chromosomes that contain more than 27,000 protein coding genes. About 15,000 genes match genes of other organisms. In addition to that the genome analysis also elucidated that a huge number of genes are based on gene duplication mechanisms. This is especially true for genes that play a role in structural complexity, sensing and response to environmental conditions and using of different resources. The sequenced genome analysis of *T. thermophila *once more illustrates the complexity of this single cell eukaryotic microorganism [2,14].

In order to get more insights into functional aspects of the *T. thermophila *genome molecular biology tools are necessary that allow the easy handling of the *T. thermophila *genes to form the basis of the postgenomic age of this model organism. The nuclear dimorphism (MIC and MAC) of the ciliates offers different possibilities of manipulating the organism's properties[15]. However, altering the phenotype ultimately needs direct or indirect genetic engineering of the vegetative MAC. The first approaches were based on the use of plasmids that take advantage of the vast amplification of the rDNA gene during *anlagen*/MAC development [16]. However, the episomal presence of these plasmids depends on the presence of antibiotics in the culture medium and the plasmids often recombinate homologously and non-directionally into the endogenous rDNA.

The stable integration of expression or knock out cassettes into the diploid MIC provides a second method to manipulate the ciliate's genome, because after conjugation of two different mating types the old MACs disintegrate and new ones form that carry the new information derived from the recombinant MIC. The advantage is that one obtains stable clones that can be crossed *via *classical Mendelian genetics to combine various properties of different *T. thermophila *strains. But this approach is elaborative and time consuming. Furthermore, it has recently been shown that scan RNAs (snRNA) derived from the old MAC epigenetically control the genome rearrangement of the new developing MAC [8,9,17]. Thus this RNAi-like mechanism may cause problems due to partial deletion of foreign expression cassettes in the developing new MAC.

So far ciliate expression vectors rely on large double rDNA origin stretches to ensure a stable propagation in *T. thermophila *cells or on large flanking integration sites of non-coding regions that are necessary for a proper and efficient homologous recombination into the gene loci of the MIC or MAC. In both cases the AT-richness of these functional DNA sequences cause problems in handling and cloning.

Recombinases like *Cre, Flp *or the λ system catalyze rearrangements of DNA at specific sequences [18-21]. This enables the insertion of mobile DNA elements into the host genome. Consequently these recombinase mechanisms were used to develop different systems that simplify the molecular genetic applications. From the technical point of view these techniques allow the flexible and fast transfer of DNA sequences from donor plasmids into multiple adequate acceptor backbones thereby circumventing restriction and ligation reactions. Thus, once inserted into a donor plasmid the selected DNA does not need to be subcloned. It is obvious that this is of high relevance in cloning very large or AT-rich sequences. In this study we present for the first time a *Cre*-recombinase dependent vector system for ciliates. It allows the independent construction of expression cassettes on the one and the preparation of acceptor vectors with integration sites on the other hand. In a second step expression cassettes can be easily shifted from the donor plasmid into various acceptor backbone constructs.

Here we describe the proof of concept of such a system for the *T. thermophila *expression host by two independent examples. We used the C-terminus of a merozoite surface antigen (MSP-1) from *P. falciparum *and a novel blasticidin resistance gene (bsdR). They were cloned into the donor vector and the gene cassettes were transferred *via *the *Cre*-recombinase into different expression vectors. Finally we showed the production of the foreign proteins in the ciliate *T. thermophila*.

## Results

The goal of this work was to establish a system that facilitates the cloning and then allows a flexible shuttling of the corresponding sequences and/or expression cassettes into the appropriate vector systems. To reach this aim we constructed a set of vectors that take advantage of a *Cre*-dependent recombinase system [18].

First we constructed the donor plasmid. We selected a pCR-TOPO vector as backbone and removed the ampicillin resistance (ampR) gene by BspHI digestion and subsequent religation of the plasmid. In a second step a 1.8 kb DNA cassette was inserted by using EcoRI sites. This artificial cassette (K42) has a modular structure and contains a histone promoter (*H4-1*), a signal peptide (encoding the first 39 aa of *P. falciparum *surface protein MSP1) fused to the EYFP reporter protein and a histidine stretch (6xH) as well as the beta tubulin terminator *(BTU2) *of *T. thermophila*. All of these DNA modules can be easily changed by using unique restriction sites. The whole cassette is flanked by *loxP *sites on the 5' and 3' ends (see Additional file 1; for basic donor plasmid see figure 5).

A chloramphenicol resistance (CmR) was inserted between the *loxP *site and the *BTU2 *terminator to reduce background clones, because this CmR is only translated in *E. coli *if a correct site-specific recombination between acceptor and donor plasmid has been occurred. However, due to the modular architecture of the pDL-plasmids also other resistance markers can be used for this purpose (*e.g*. tetracycline, zeocin etc.). In a next step, the *sacB *marker gene was inserted into the intermediate vector. The *sacB *gene product metabolises sucrose into levansucrose, a toxic substance for *E. coli *cells. The parallel usage of the Cm resistance (selection) and the *sacB *(counter-selection) gene strongly inhibits the presence of non-recombinant clones. Finally, we replaced the Enhanced Yellow Fluorescence Protein (EYFP) cDNA by the MSP1_19 _cDNA from *P. falciparum *or the blasticidin resistance gene (bsdR) to demonstrate that the whole system facilitates cloning and expressing foreign genes like previously shown for other host systems *e.g*. arabidopsis.

**Figure 1:**
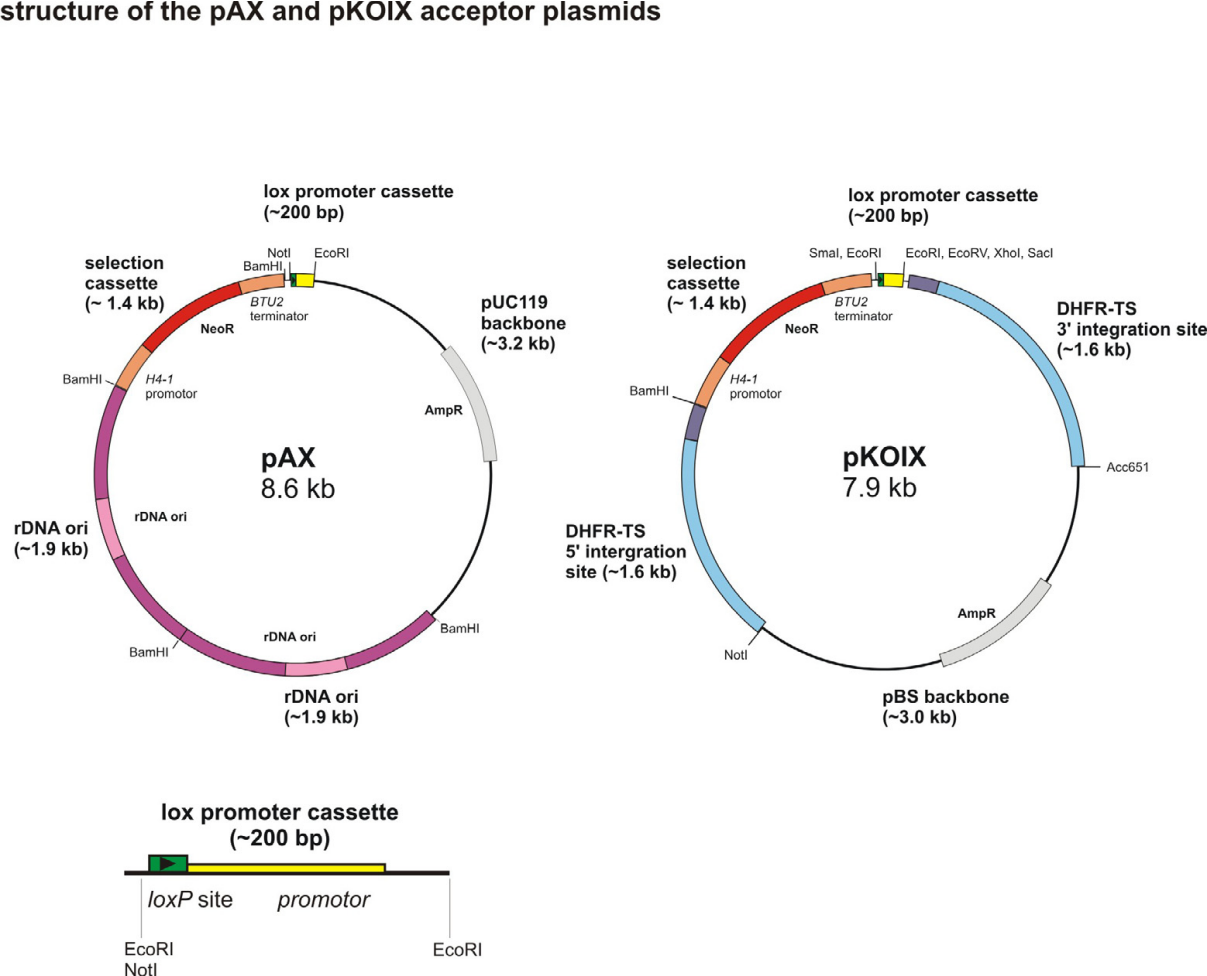
**The acceptor vectors pAX and pKOIX**. Structure of pKOIX and pAX acceptor plasmids. The loxprom site that consists of a *loxP *site (green) and the bacterial promoter (yellow) was inserted into pH4T2 by using the NotI and EcoRI and into pKOI by EcoRI restriction sites, respectively. The arrow head in the *loxP *sites indicates the *loxP *orientation. The gray/black parts correspond to the backbone sequences that are necessary for propagation in *E. coli*, and the red part of both acceptor vectors illustrate the *neo2 *selection cassette for selection in *T. thermophila *by paromomycin. The purple colored part of pAX illustrates the AT-rich double rDNA *ori *which is necessary for the propagation in *T. thermophila*. The blue part in pKOIX shows the AT-rich 5' and 3' integration sequences (bright blue, up/downstream and dark blue coding sequences). They are part of the endogenous DHFR-TS gene locus.(Bottom: detail of loxprom site).

Recently, we described the bifunctional dihydrofolate reductase and thymidylate synthase (DHFR-TS) of *T. thermophila*. Both enzyme activities play a crucial role in DNA synthesis. The loss of these essential activities can be used as an auxotrophic marker in *T. thermophila*. We developed a vector system that combines the *knock out *of the endogenous DHFR-TS gene with the *knock in *of an expression cassette that encodes a foreign gene (pKOI) [23]. Appropriate acceptor vectors for the *T. thermophila *system were created by cloning the *loxP-*promoter site (loxprom) into this pKOI vector backbone as well as into a previously described rDNA based episomal plasmid (pH4T2). The new vectors were named into pKOIX (pKOI backbone) and pAX (pH4T2 backbone). A scheme of the acceptor vector structure is given in figure 1.

The final expression vectors pAX-MSP1_19_/pAX-bsdR and pKOIX-MSP1_19_/pKOIX-bsdR were generated *via *the novel recombinase mechanism. In general, a mixture of 100 ng of donor and 100 ng of acceptor plasmid yielded 30 to 50 initial positive clones that were able to grow on LB-agar plates supplemented with both chloramphenicol and ampicillin.

**Figure 2:**
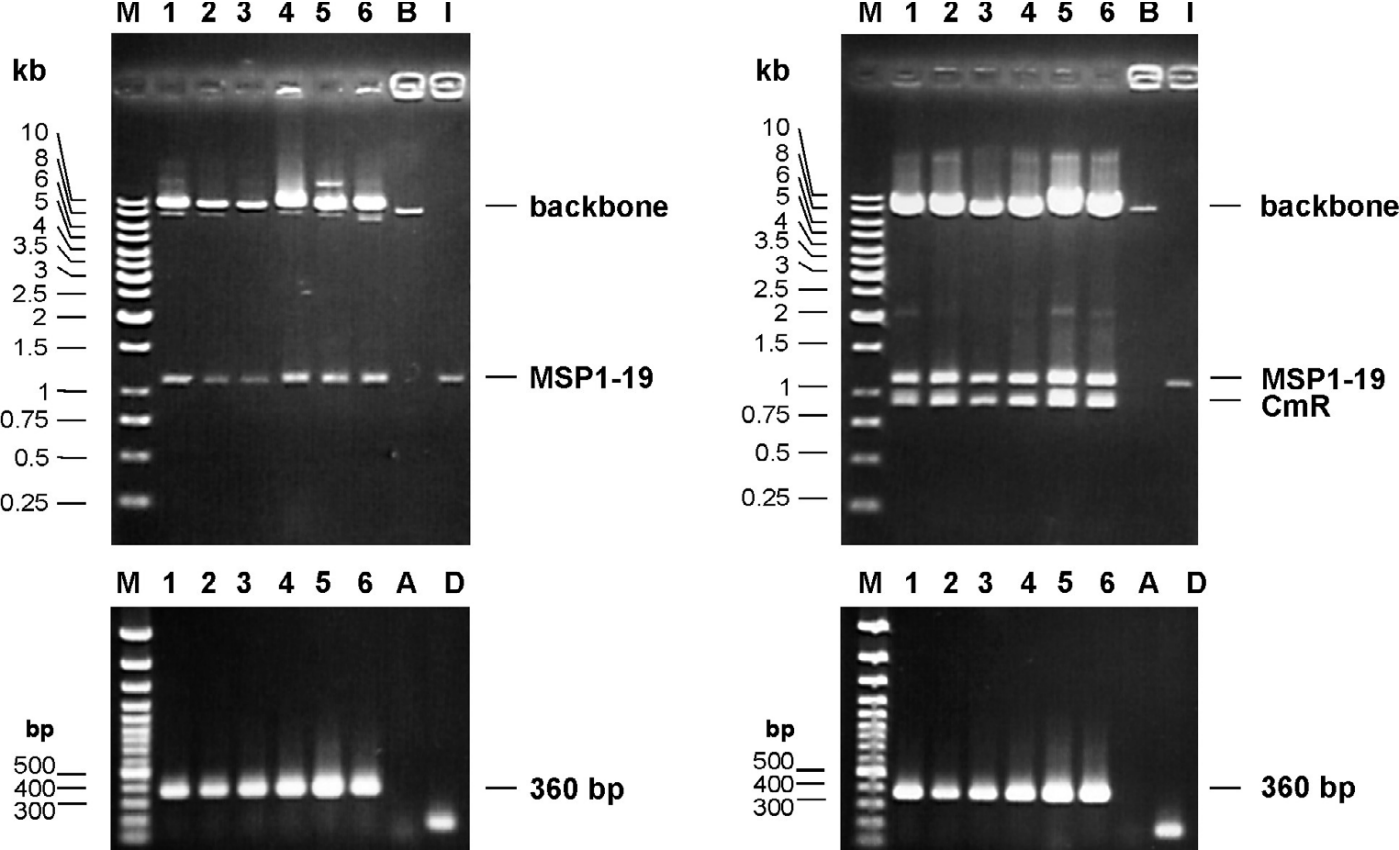
**Analysis of the Cre-recombinase reaction with novel donor and acceptor plamids**. Results of the *Cre*-recombinase reactions using pKOIX, pAX, and pDL-MSP1_19_, respectively. *Left column: *Analysis of recombinant pAX-MSP1_19 _clones. *Top*: The restriction analysis of recombinant pAX clones with XhoI and SacI and are shown. The analyzed positive clones (1–6) have an 8.6 kb backbone and a 1.2 kb insert. The vector backbone without the insert (control B) and the donor plasmid insert (MSP1_19_) are shown in lanes B and I. *Bottom*: The diagnostic PCR confirms that all analyzed clones are positive, because the amplification of the specific 360 bp fragment is only possible in recombinant clones. The acceptor control (A) is negative and the fragment in the donor control (D) is an unspecific artefact (200 bp). *Right column: *The analogous approach as shown in the left column with the acceptor plasmid pKOIX instead of pAX. The analyzed positive clones carry a 7.9 kb backbone and the 1.2 kb insert. The additional band at 1.0 kb is due to a SacI site in the Cm resistance. Lanes B and I show the used backbone (pKOIX) and the 1.2 kb insert (MSP1_19_). The diagnostic PCR leads to the specific 360 bp fragment like in the pAX-MSP1_19 _approach. The 200 bp fragment in the donor control, (D) is an unspecific artefact. The acceptor control (A) is negative.

We picked six clones of the MSP1_19 _recombinase reactions and analyzed them by diagnostic PCR and restriction analysis to test the efficiency of the novel recombinase approach. All of the analyzed clones were positive (6/6). This finding was independent of the used acceptor plasmid. The recombinant pAX- as well as pKOIX-plasmids carry complete expression cassettes and an all complete plasmid backbone. The results are shown in figure 2. The left column illustrates the results using the pAX and right column the pKOIX backbone. Positive clones were analyzed by restriction analysis (XhoI and SacI) and diagnostic PCR (360 bp fragment in positive clones), verifying a correct recombinase event. In general nearly all clones (80–100%) obtained were positives and have a complete backbone. We obtained such quotes in all performed *Cre*-recombinase reactions (data not shown).

We compared the recombinase efficiency of generating recombinant expression plasmids standard cloning techniques. The pDL-MSP1_19 _(see Additional file 2) was digested with NotI and SacI and the corresponding insert (*H4-1*-MSP1_19_-*BTU*2) was ligated into the pre-cut pH4T2 vector to obtain an rDNA-based MSP1_19 _expression plasmid. An analogous approach was done with the pKOI plasmid. Eight clones were randomly picked and analyzed by restriction analysis. All of them (8/8) were negative and most of the pH4T2 backbones were degraded or fragmented during the ligation, transformation selection and propagation procedure. The supplementary figure 1 illustrates a typical result of such an approach. In most cases only 2–3% (one of 30 to 50 clones) carries the complete expression cassettes in a complete plasmid.

**Figure 3:**
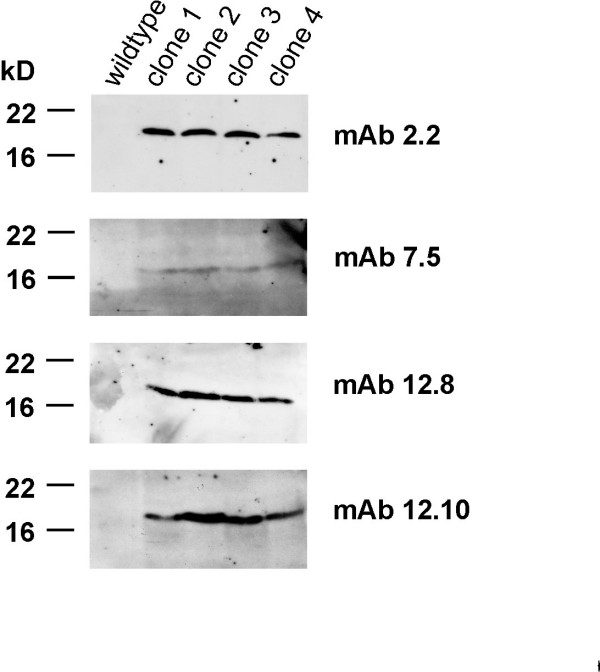
**Expression of the 19 kDa C-terminus of MSP1 in *T. thermophila***. Aliquots (extract of 50,000 cells) of a non-transformed wildtype and cells of transformed clones (clones 1–4) that were transformed with pKOIX-MSP1_19 _construct were separated on 15 % SDS-PAGE. The recombinant 19 kDa fragment of MSP1 could be detected in all clones (1–4) with four independent monoclonal antibodies (mAbs 2.2, 7.5, 12.8 and 12.10) all derived against the C-terminus of MSP1 of *P. falciparum*. No signal could be observed in the wildtype negative control. The monoclonal antibodies were kindly provided by Prof. J. McBride (Edinburgh, UK).

Extracts of cells that were transformed with the pKOIX-MSP1_19 _plasmid were analyzed for expression of a 19 kDa protein fragment of the MSP1 protein. We used cell extracts of four independent stably transformed strains and compared them to the non-transformed 1868/7 wildtype strain. In all tested cell extracts the recombinant 19 kDa fragment of MSP1 (MSP1_19_) could be detected by the specific monoclonal antibodies (mAb2.2, mAb7.5, mAb12.8 and mAb12.10 were kindly provided by Prof. McBride, Edinburgh, UK) [26,27]. In the wildtype negative control no signal could be found. The results are summarized in figure 3. In previous expression experiments we could also demonstrate that the rDNA plasmid is capable of expressing the MSP1 C-terminus (data not shown).

**Figure 4:**
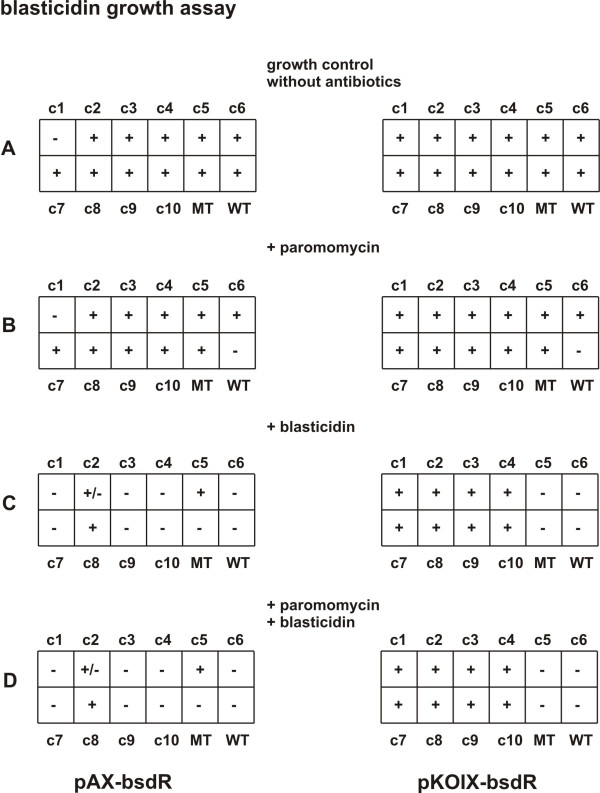
**Blasticidin growth assay**. We created a donor plasmid – pDL-bsdR – that encodes the blasticidin resistance gene under the control of the *H4-1 *promoter and the *BTU2 *terminator (see figure 5B). It has a similar structure as the previously described *neo2 *cassette (resistance against paromomycin) that is also present in the here used acceptor plasmids pAX and pKOIX. Using the *Cre*-dependent recombinase we generated the expression plasmids pAX-bsdR and pKOIX-bsdR and transformed *T. thermophila *strains. Blasticidin resistance assay: *left column*: clones of cells transformed with pAX-bsdR, *rightcolumn: *clones of cells transformed with pKOIX-bsdR. Several independent clones (c1 to c10) were tested for bsd resistance. As controls we used the wildtype strain 1868/7 (WT) and a mock transformant (MT) that only carried the *neo2 *resistance gene **A: **growth control of clones in SPP-medium without antibiotics; **B: **same clones as presented in A selected in SPP-medium with 400 μg/mL paromomycin after 5–10 days **C: **same clones as in B, but cultivated for 3–5 days in SPP-medium with 100 μg/mL blasticidin; **D: **identical clones as in B/C cultivated in SPP-medium for 3–5 with both antibiotics, paromomycin (400 μg/mL) and blasticidin (100 μg/mL). **+ **strong growth of clones. **+/- **cells alive, less growing. - no growth, cells died within 2–3 days.

We attempted to confirm the *Cre*-recombinase dependent cloning by using a second independent expression module. Therefore pAX and pKOIX constructs that carry the bsdR expression cassette (pAX-bsdR, pKOIX-bsdR) were transformed into conjugating and vegetative *T. thermophila *wildtype strains according to protocols previously described. The transformants (clone1- clone10) were cultivated in SPP-medium supplemented with thymidine and increasing concentrations of paromomycin (figure 4B) to ensure a stable propagation of the clones. The same clones were cultivated in SPP-medium without antibiotics (figure 4A). In a second step we performed a blasticidin growing assay and switched the antibiotic from paromomycin to blasticidin (figure 4C) or applied both antibiotics in parallel. In all experiments the wildtype (WT) that did not contain a resistance gene died within 2–5 days. As expected the mock transformant (MT) that only carried the *neo2 *cassette (resistance against paromomycin) died when blasticidin was added to the SPP-medium (bsd antibiotic control).

Interestingly, we observed that the pKOIX-bsdR transformants were more stable when compared to the pAX clones (figure 4D). Eight out of the ten analyzed independent pKOIX-bsdR clones are resistant against both antibiotics. In contrast to that only ~30% of the pAX clones (3/9) displayed both resistances. This is probably due to recombination events between the rDNA plasmids backbones and/or due to the very similar architecture of the *neo2 *and bsdR resistance cassettes (see figure 1). In summary these results illustrate that the *Cre*-dependent modular donor plasmids in combination with the recently described *knock out/knock in *concept (pKOIX) provides an easy and sustainable system to establish a resistance testing tool.

## Discussion

Protozoan and functional genomics are an exciting research area. More and more genomes of eukaryotic microorganisms have already been completely sequenced (e.g. *Plasmodium falciparum*, *Tetrahymena thermophila*, *Paramecium tetaurelia*) or will be available soon (*e.g*. genomes from species of *Toxoplasma *or *Entamoeba*) [2,28-30]. This allows new insights into evolutionary mechanisms as well as the discovery of new biochemical pathways or the identification of promising vaccine candidates against pathogenic protozoans.

The recently characterized genome of the ciliate *Paramecium tetaurelia *for example elucidated that three successive whole genome duplications lead to nearly 40,000 genes, illustrating that these mechanism allows a an excellent adaptation to environmental conditions [29].

However, functional genomics and subsequent proteomic studies require tools that allow the analysis and manipulation of certain genes of interest. For the most common model organisms and expression systems like mammalian cell lines, *Drosophila*, yeast or *E. coli *these tools have been developed and optimized for decades. In contrast to this some tools are lacking to deal with unusual properties and pitfalls of unusual organisms. AT-rich genomes for example cause difficulties in handling the DNA sequences. Also the AT-richness of the *T. thermophila *genome causes the main challenge in altering the ciliates phenotype. Previously described episomal expression plasmid consist of a pUC backbone that enables propagation in *E. coli*, two 1.9 kb parts forming the rDNA *ori *and the *neo2 *cassette that allows the selection of the transformed ciliates. The empty vector is sized about 8.4 kb. Especially the two rDNA origins are (3.8 kb) AT-rich sequences [22]. These sequences are probably one reason why this vector tends to recombinate into the highly amplified endogenous rDNA chromosomes of the host cells.

Recently, we developed a knock out/knock in system (pKOI) that is based on the stable integration into the endogenous gene locus of the DHFR-TS. The main advantage of this concept is that a stable *knock out *can be monitored by the complete loss of the DHFR-TS activity, resulting in an auxotrophy for thymidine. Thus this marker system allows the propagation of recombinant *T. thermophila *cells without rDNA *ori *sequences [23]. However, large AT-rich stretches are necessary to ensure good integration efficiency by homologous recombination. In the case of the pKOI constructs a 1.6 kb regions of the 5'- and a 1.6 kb 3'-region of DHFR-TS gene have been added to the vector backbone. The uptake of ligation reactions and the subsequent amplification in *E. coli *often resulted in reduced and fragmented backbones and the loss of the expression cassettes. This indicates that the AT-rich DNA of the double rDNA or of the integration sites is responsible for the described problems. There is a demand for simplification of the genetic manipulation of AT-rich protozoans. Therefore both available vector systems were optimized.

Creating and tuning of expression cassettes in a small and flexible donor vector and the subseqeuent construction of final expression vectors by an easy and robust shuttling of the expression module provided a solution to this problem. The constructed donor vector possesses a modular structure and is small sized. It lacks AT-rich sequences like rDNA or integrative sites. Thus the gene of interest as well as signal peptides, promoter and terminator sequences can easily be substituted via unique restriction sites. This offers the possibility to establish simple test systems like the here shown resistance gene test system. Furthermore, different constitutive, cell cycle dependent or inducible promoter sequences can be combined to various genes of interest.

The whole DNA cassette of the donor vector is flanked by *loxP *sites to enable the *Cre*-dependent site-specific transfer into appropriate acceptor plasmids.

As acceptor plasmids we used both, the backbone of the episomal rDNA plasmid pH4T2 and the recently described pKOI backbone. Up to now, these concepts and the paclitaxel system developed by Gaertig et al. are the only known expression vector concepts that are available for the *T. thermophila *system [12,23]. We did not only observe a complete transfer but also an expression of the encoded genes of the shuttled expression module (bsd resistance gene and the C-terminus of the MSP1 protein), illustrating the high efficiency of the new system.

The ciliates are one of three evolutionary lineages that make up the alveolates. The two further groups are dinoflagellates and apicomplexans. Especially the endoparasitic apicomplexans contain a number of human and animal pathogens (*e.g*. the genera *Plasmodium, Toxoplasma*, *Cryptosporidium *(for details see the apicomplexans database [31]). The most important protozoans from this apicomplexans group are *Plasmodium *species that causes malaria.

Intriguingly, the genome of *Plasmodium falciparum *is the most AT-rich genome known so far (for details see Plasmodium database [30,32]). This illustrates that the handling of (large) AT-rich DNA sequences is not a problem limited to *T. thermophila *applications. However, we and others demonstrated that the *T. thermophila *expression system is able to express proteins from the malaria parasite *Plasmodium faciparum*, suggesting that this "distant cousin" can serve as an expression system for proteomic applications of other protozoans [33]. Furthermore, the here shown vectors can be modified to enable an adaptation to optional requirements. For example, the DHFR-TS integration sites of pKOI can be substituted by DNA sequences of other species to allow an integration into other host systems (e.g. *Plasmodium *or *Paramecium *species). Thus the here presented powerful molecular biological tool allows a more flexible and easy handling of DNA sequences and might reveal a concept that can easily be transferred to other protozoan host systems.

## Conclusion

The here presented *Cre*-dependent recombinase system is the first one that has been established for a protozoan system. It allows a facilitated shuttling of DNA into expression vectors and thereby an easier handling of these AT-rich DNA sequences.

As the concept can easily be adapted to further unicellular eukaryotes the whole system provides a powerful molecular biology tool in protozoan research.

## Methods

### Constructs

#### Donor plasmid

The construction of the donor plasmid was done in several steps. Firstly, a pCR-TOPO backbone was restricted with BspHI and re-ligated to eliminate the ampicillin-resistance (ampR). Secondly, this smaller sized backbone that only carries the remaining kanamycin resistance (kanR) was digested with EcoRI to insert the K42 cassette (made by Geneart, Germany). This artificial K42 expression cassette encodes the first 39 amino acids of the MSP1 sequence, fused to enhanced yellow fluorescence protein (EYFP) and to a histidine tag. The expression of this gene cassette is controlled by a histone promoter *(H4-1) *and a beta tubulin terminator *(BTU2)*. The whole K42 cassette is flanked by two *loxP *sites ("floxed") of the same orientation. In the third step a chloramphenicol resistance gene (CmR) was amplified with the primers CmRF: (5'-TTTactagtttaaacATAACTTCGTATAATGTATGCTATACG-3') and CmRR (5'-TTTgagctcggccggccAAATTACGCCCCGCCCTGCCACTC-3') and inserted into the K42 sequence by using SpeI and SacI. The CmR gene is localized between the *BTU2 *terminator and the down stream *loxP *site. In a fourth step the *sacB *gene from *Bacillus subtilis *was amplified by using the primers pair sacBF/R (5'-TTactagtACATATACCTGCCGTTCACTATTATTTAGTG-3') and (5'-TTactagtGGCATTTTCTTTTGCGTTTTTATTTGTTAACTGTTAATTGTCC-3'). This sequence was inserted by using a unique SpeI site of the intermediate donor vector site of the third step. The *sacB *sequence is necessary to allow a counter selection by adding sucrose to the LB-agar plates. For amplification of the CmR and *sacB *sequences a pDNR was taken as template (data not shown). The combination of the CmR and *sacB *gene is similar to the previously established Creator (BD Clontech, Heidelberg) concept. Finally, the intermediate vector pDL-K42 was digested with EcoRV and BglII to replace an EYFP insert by a cDNA encoding the 19 kDa C-terminus of the merozoite surface antigen 1 of *Plasmodium falciparum *(MSP1_19_) and the cDNA of the blasticidin resistance gene (bsdR), respectively. The bsdR cDNA was amplified using the primers bsdF/R (5'-gatatcATGGCCAAGCCTTTGTCTCAAG-3') and (5'-ttagatct TCAGCCCTCCCACACATAACCAGAGG-3'). The MSP1_19 _cDNA was amplified from a genomic DNA preparation of *P. falciparum *3D7 (kindly provided by Prof. Lanzer Heidelberg, Germany) by using the primers MSP1_19_-F (5'-AACATTTCACAACACCAATGCG-3') and MSP1_19_-R (5'-AAggatccTCAAATGAAACTGTATAATATTAACATGAG-3').

Both cDNAs were restricted (BglII or BamHI) to insert the fragments into the EcoRV/BglII pre-cut, linearised pDL-K42 vector. All constructs were verified by sequencing (Carpegen, Muenster, Germany).

#### Acceptor plasmids

##### Episomal acceptor plasmid

We amplified the lox-promoter cassette by using the primers loxprom F/R: (5'-AGTCTgaattcACGTCAGGTGGCACTTTTC-3') and (5'-TTgaattcgcggccgcATAACTTCGTATAG-3'). As template we used the lox-promoter site of the acceptor vector pLP GADT7 (BD Clontech, Heidelberg). The small letters correspond to the used restriction sites within the sequence. The corresponding loxprom PCR fragment was digested with EcoRI and NotI and cloned into a previously described double rDNA *ori *based pH4T2 shuttle plasmid [22]. This new acceptor plasmid was named pAX.

##### Integrative acceptor plasmid

The recently described knock out construct pKOI that allows the integration into the dihydrofolate reductase thymidylate synthase (DHFR-TS) gene locus of *T. thermophila *was used to construct a second acceptor vector [23]. We used the unique EcoRI site to insert the lox-promoter site. The *loxP *orientation promotes a site-specific insertion in the same orientation as the *neo2 *resistance cassette of pAX and pKOIX. Details on primers donor and acceptor sequences and the constructs are available at Cilian AG (Muenster, Germany).

### Cre-recombinase reaction and selection in *E. coli*

Donor plasmids pDL-bsdR and pDL-MSP1_19 _and acceptor plasmids pKOIX and pAX were amplified in LB-medium containing kanamycin or ampicillin, respectively. The recombinase reactions were performed as follows: Aliquots of 100 ng donor and 100 ng acceptor plasmids were mixed in reaction buffer. *Cre*-recombinase was added and samples were incubated for 25 min at 25°C. The reaction was stopped by heat inactivation for 10 min at 70°C. Aliquots of the reaction were purified by using the Montage Kit (Millipore, Schwalbach) and transformed into *E. coli *strain DH10B (Invitrogen, Heidelberg). Positive clones were selected on LB-agar plates supplemented with ampicillin, chloramphenicol and 7% sucrose. Recombinant pKOIX and pAX plasmids were propagated in LB-medium containing ampicillin. First the clones were analyzed by diagnostic PCR by using the primers PCP1 (5'-GCTCACCGTCTTTCATTGCC-3') that binds in the Chloramphenicol resistance gene and PCP2 (5'-TCCGCTCATGAGACAATACC-3') that binds in the prokaryotic Chloramphenicol promoter region. Both elements are only connected by a successful recombinase reaction, leading to a specific PCR product (ca. 360 bp). Clones were analyzed using the restriction enzymes XhoI and SacI to verify whether or not the backbone was complete.

### Ciliate strains, cultivation and transformation

*Tetrahymena thermophila *strains B 1868/4, B 1868/7 and B 2068/1 were cultivated in skimmed milk medium (2% skimmed milk, 0.5% yeast extract, 0.1% ferrous sulphate chelate solution and 1% glucose) in SPP-medium (0.5% proteose peptone, 0.5% yeast extract, 0.1% ferrous sulphate chelate solution and 1% glucose) or in modified CDM (modified from Hellenbroich et al [24]) medium as described previously [23,25]. We used vegetative growing non-conjugating *T. thermophila *strains. The transformation of the *T. thermophila *cells was performed as described previously. Transformed cells were distributed on 96 well plates. Positive individual clones were isolated (single cell isolation) and further cultivated in 24 well plates.

### Selection, allelic assortment and DHFR-TS knock out assay

*T. thermophila *cell proliferation assay: For the first 16 h after biolistic bombardment transformants were grown in skimmed milk medium. After that transformed cells were grown on SPP-medium with increasing concentrations of paromomycin (from 100 μg/mL to 1000 μg/mL) to support the allelic assortment process. After 2–4 weeks each clone was cultivated on CDM replica plates with or without thymidine (10 mg/mL). Functional DHFR-TS knock out clones are only able to grow in CDM medium supplemented with thymidine. The viability of the DHFR-TS knock out strains was monitored by determining the growth kinetic as previously described in more detail [23].

### Blasticidin growing assay

Clones that were transformed with pAX-bsdR or pKOIX-bsdR plasmids were first selected in SPP-medium supplemented with paromomycin and thymidine. After that the presence of the bsd resistance cassette was verified by adding 100 μg/mL blasticidin to the CDM/SPP-media. Positive clones are resistant against the bsd addition to the culture medium, negative clones died within two to three days. Both pAX-bsdR as well as pKOIX-bsdR clones are able to grow in SPP/CDM-media with both antibiotics in the medium (400 μg/mL paromomycin and 100 μg/mL blasticidin).

### SDS-PAGE and Western blot

SDS-PAGE and Western blot analysis were done as previously described [23,25]. Briefly, aliquots (50,000 cells) of transformed cells were resuspended in sample buffer boiled for 10 min and separated on 15% SDS-PAGE. The gels were blotted onto nitrocellulose membranes and blocked of 1 h at room temperature (or at 4°C overnight) in PBS containing 0.05% Tween 20 and 5% skimmed milk (PBS-TM). The expression of recombinant MSP1_19 _in transformed ciliates was detected by specific monoclonal anti MSP1_19 _antibodies kindly povided by Prof. McBride (Edinburgh, UK). The monoclonal antibodies were diluted 1:100 in PBS-TM and incubated for 1 hour at room temperature. After washing with PBS/T for 30 min we applied the second antibody (HRP-conjugated goat anti mouse serum; dilution 1:1000) in PBS-TM. The blots were developed by chemiluminescence using SuperSignal West Femto Max Sensitivity Substrate (Pierce Biotechnology) in combination with conventional X-ray film development.

## Authors' contributions

Most experiments, the concept and the manuscript were made by TW, AR generated the intermediate plasmids and UB transformed the ciliates and selected the positive clones. LH participated in construction of the pKOI backbone and MWWH in the conceptual work. Vectors and strains can be made available upon request from MWWH. All authors read and approved the final manuscript.

## Supplementary Material

**Figure 5:**
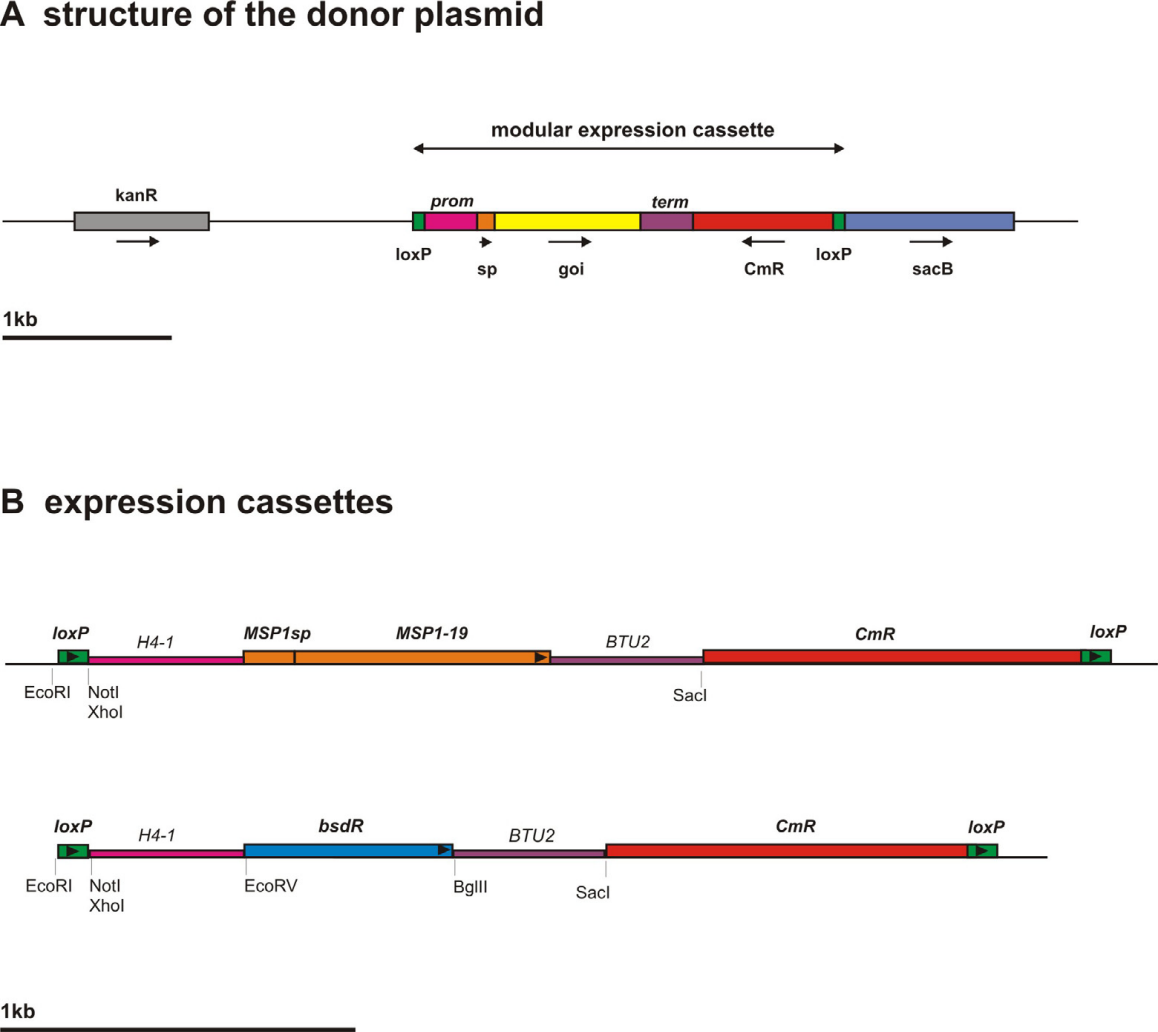
**Structure of the used donor plasmids and expression cassettes**. **A: **Scheme of the basis donor plasmid (linear structure) with its modular structure. The cassette allows the simple substitution of promoter, signal peptide (sp) gene of interest and terminator DNA sequences without loosing the flanking *loxP *sites due to unique restriction sites. **B: **"Floxed" (flanked by *loxP*) expression modules were used that encode an expression cassette for the MSP1 C-terminus gene (top) and the blasticidin resistance (bsdR, bottom) gene, respectively. All expression cassettes are flanked by a histone promoter (*H4-1*) and a beta tubulin terminator (*BTU2*).

Additional File 1 Construction of the first donor plasmid. **A: **Scheme of the used modular artificial cassette K42. It allows the substitution of promoter, gene of interest and terminator sequences without losing the flanking *loxP *sites. **B: **The "floxed" artificial sequence was inserted into a pCRTOPO backbone, lacking the ampicillin resistance gene. In the next steps a chloramphenicol resistance and a *sacB *counter-selection cassette were added. This basic donor plasmid was used to replace the EYFP cDNA (see figure 5). Click here for file

Additional File 2 Recombination and fragmentation of large AT-rich plasmids. This figure illustrates the undesired recombination events that lead to fragmented plasmids during the standard cloning procedure (ligation, transformation, selection and propagation) in *E. coli*. M: marker; 1 kb ladder (generuler, MBI Fermentas, 1–8: Analyzed clones; B/I: backbone DNA (8.4 kb) and insert DNA (ca. 1.2 kb) that was used for the ligation reaction. Click here for file
