# Supplementary figures and images for: A recombinase system facilitates cloning of expression cassettes in the ciliate Tetrahymena thermophila
Source: BMC Microbiol. 2007 Mar 1;7:12. doi: 10.1186/1471-2180-7-12 (PMC1839094; doi:10.1186/1471-2180-7-12)

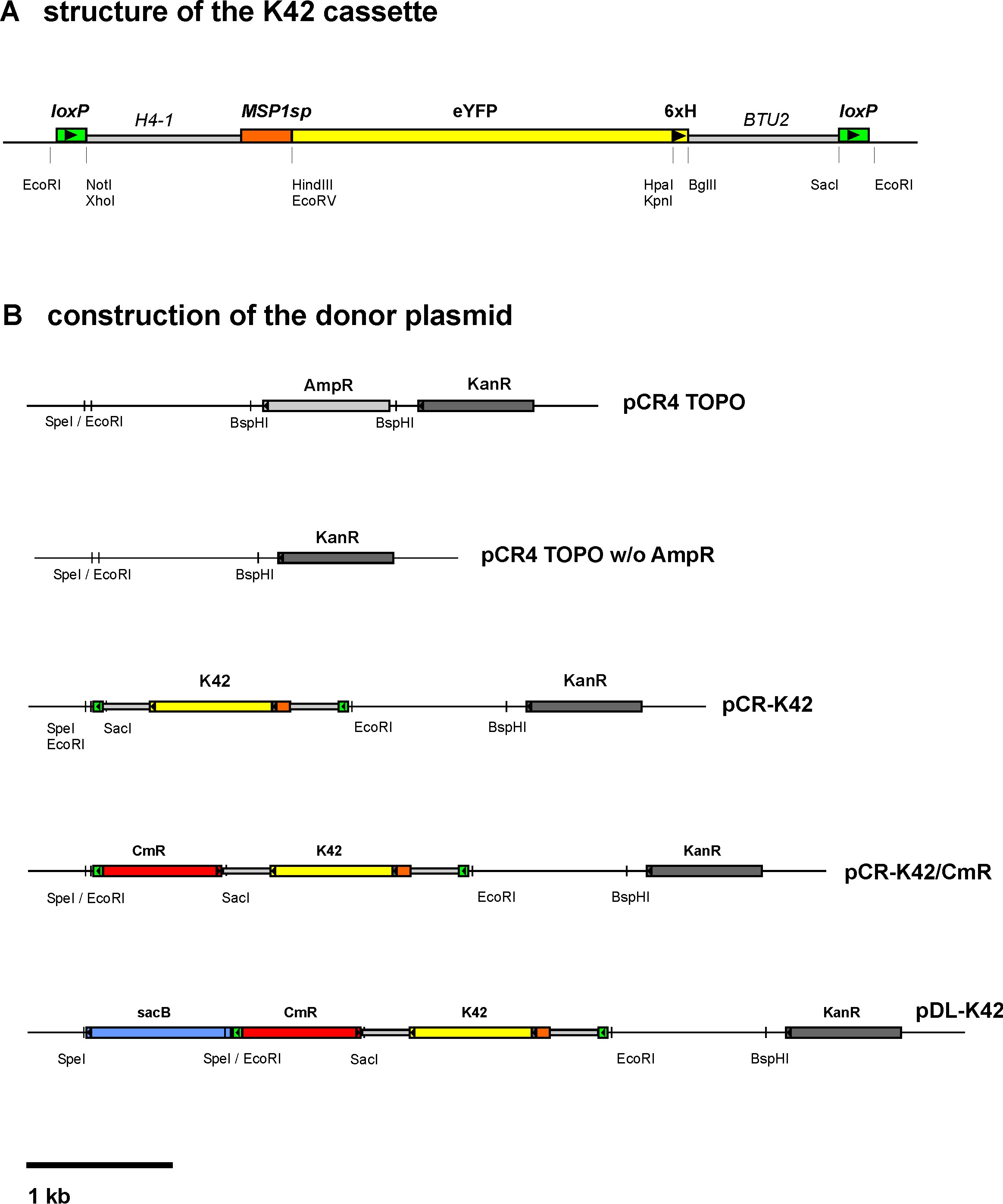

Supplement: Additional File 1 — Construction of the first donor plasmid. A: Scheme of the used modular artificial cassette K42. It allows the substitution of promoter, gene of interest and terminator sequences without losing the flanking loxP sites. B: The "floxed" artificial sequence was inserted into a pCRTOPO backbone, lacking the ampicillin resistance gene. In the next steps a chloramphenicol resistance and a sacB counter-selection cassette were added. This basic donor plasmid was used to replace the EYFP cDNA (see figure 5). [file 1471-2180-7-12-S1.jpeg]

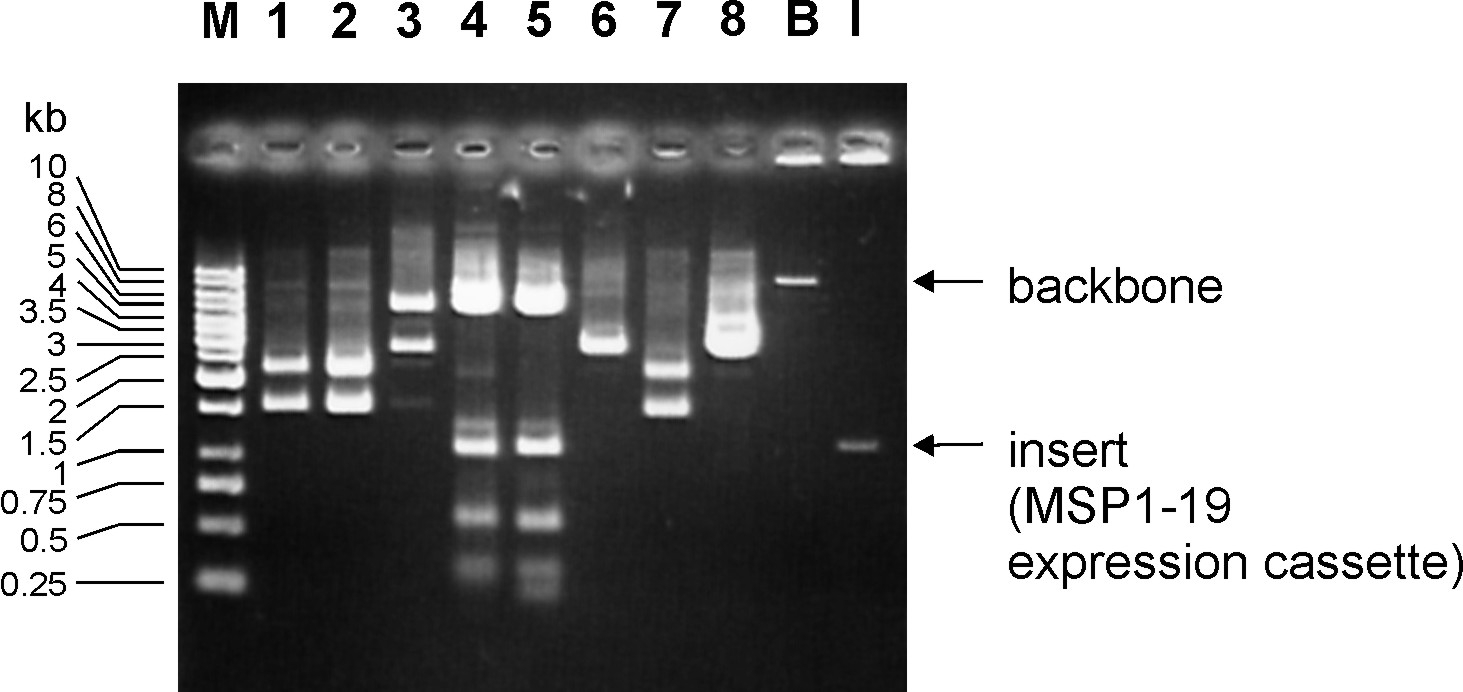

Supplement: Additional File 2 — Recombination and fragmentation of large AT-rich plasmids. This figure illustrates the undesired recombination events that lead to fragmented plasmids during the standard cloning procedure (ligation, transformation, selection and propagation) in E. coli. M: marker; 1 kb ladder (generuler, MBI Fermentas, 1–8: Analyzed clones; B/I: backbone DNA (8.4 kb) and insert DNA (ca. 1.2 kb) that was used for the ligation reaction. [file 1471-2180-7-12-S2.jpeg]
